# β-arrestin1 at the cross-road of endothelin-1 signaling in cancer

**DOI:** 10.1186/s13046-016-0401-4

**Published:** 2016-07-29

**Authors:** Laura Rosanò, Anna Bagnato

**Affiliations:** Preclinical Models and New Therapeutic Agents Unit, Translational Research Functional Departmental Area, Regina Elena National Cancer Institute, Via Elio Chianesi, 53, 00144 Rome, Italy

**Keywords:** Endothelin, Endothelin receptors, Cancer, β-arrestin, G-protein coupled receptors

## Abstract

The advent of targeted therapeutics in human cancer has begun to find novel druggable targets and, in this context, the endothelin-1 receptor (ET-1R), namely ET_A_ receptor (ET_A_R) and ET_B_ receptor, among the GPCR family represents a class of highly druggable molecules in cancer. ET-1R are aberrantly expressed in human malignancies, potentially representing prognostic factors. Their activation by ligand stimulation initiate signaling cascades activating different downstream effectors, allowing precise control over multiple signaling pathways. ET-1R regulates cell proliferation, survival, motility, cytoskeletal changes, angiogenesis, metastasis as well as drug resistance. The molecular events underlying these responses are the activation of transcriptional factors and coactivators, and downstream genes, acting as key players in tumor growth and progression. ET-1R represent crucial cancer targets that have been exploited for ET-1R therapeutics. Importantly, efforts to explore new information of ET_A_R in cancer have uncovered that their functions are crucially regulated by multifunctional scaffold protein β-arrestins (β-arrs) which orchestrate the multidimensionality of ET_A_R signaling into highly regulated and distinct signaling complexes, a property that is highly advantageous for tumor signaling. Moreover, the role of β-arr1 in ET-1 signaling in cancer highlights why the pleiotropic effects of ET-1 and its dynamic signaling are more complex than previously recognized. In order to improve therapeutic strategies that interfere with the widespread effects of ET-1R, it is important to consider antagonists able to turn the receptors “off” selectively controlling β-arr1-dependent signaling, highlighting the possibility that targeting ET_A_R/β-arr1 may display a large therapeutic window in cancer.

## Background

The endothelin (ET) family comprises three isoforms of small peptides, ET-1, ET-2 and ET-3, products of different genes [1]. ET-2 and ET-3 differ from ET-1 only in two and six amino acid residues, but are separate gene products, with tissue-specific expression. They are synthesized as precursor proteins of about 200 amino acid residues in size, pre-pro-ETs, and then cleaved by a neutral endopeptidase to form an inactive precursor of about 37–41 amino acids, big ETs. Finally, a family of ET-converting enzymes operates the conversion of big ET to the mature 21 amino acids peptides [1]. The levels of prepro-ET-1 are modulated predominantly at the level of transcription, through the involvement of several transcription factors, including activator protein 1, nuclear factor kappa B (NFkB), forkhead box protein O1 (FOXO1), hypoxia-inducible factor 1α (HIF-1α), and GATA2, although both physical and chemical stimuli contribute to alterations in levels of preproET-1 mRNA in physiologic and pathophysiological conditions [1, 2]. In last years, a host of epigenetic modifications have been discovered that allow for precise tuning of DNA exposure and read-out in ET system regulation, also in cancer. Indeed, in colon cancer, hypermethylation of ET-2 and ET-3 genes results in the epigenetic inactivation of ET-2 and ET-3 mRNA and corresponding protein [2]. Recent data highlighted a role of microRNAs (miRNA) to directly target the 3’UTR of ET-1 mRNA for turnover and subsequently to regulate its expression [3], and the list of miRNAs that may modulate ET-1 expression in different cancers, including hepatocarcinoma and gastric cancer, is growing [4, 5].

**Fig. 1:**
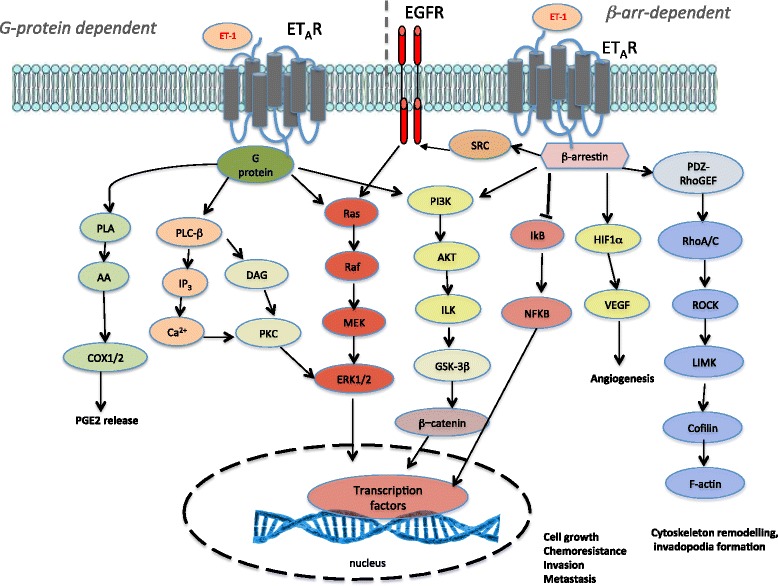
Signaling pathways activated by ET-1 in cancer. The endothelin- 1 receptor (ET-1R) is a G-protein coupled receptor, that upon agonist binding, results in the activation of G-protein-dependent primary effectors including phospholipase Cβ (PLCβ), which cleaves phosphatidylinositol- 4,5-bisphosphate (PtdIns(4,5)P2) into diacylglycerol (DAG) and inositol triphosphate (IP3), leading to calcium mobilization and protein kinase C (PKC) activation, and downstream activation of MAPK family members, including ERK1/2. At the same time, ET-1R activation stimulates Ras/Raf/MEK activation, converging on ERK1/2 signaling. Moreover, ET-1R receptor stimulation activates phospholipase A (PLA) and downstream arachidonic acid (AA) and cyclooxygenase-1 (COX-1) and COX-2, leading to prostaglandin E2 (PGE2) release, as well as phosphatidylinositol-3-kinase (PI3K), leading to the activation of AKT, integrin-linked kinase (ILK) and glycogen synthase kinase (GSK)-3β, which stabilizes β-catenin. Notably, ET-1R can also signal via β-arrestin1 (β-arr1) to activate ERK1/2 and PI3K/AKT/β-catenin signaling. β-arr1 also controls the crosstalk between ET-1R and epidermal growth factor receptor (EGFR) through the recruitment and activation of c-Src, resulting in downstream pathway activation. Through β-arr1, ET-1 activates also nuclear factor-kB (NF-kB) signaling via inhibition of NF-kB inhibitor (IkB), resulting in the dissociation and subsequent nuclear localization of active NF-kB. On the other hand, β-arr1 mediates ET-1-induced hypoxia-inducible factor 1α (HIF-1α) activity promoting vascular endothelial growth factor (VEGF) release. Moreover, ET-1R activates PDZ-RhoGEF leading to Rho-A and -C GTPase activation, initiating Rho-dependent signaling events through RHO-associated coiled-coil containing protein kinase 1 (ROCK1), LIMK activation, causing cofilin inhibition and cytoskeletal remodelling. The cooperation of these intracellular signaling pathways promote cell growth, chemoresistance, angiogenesis, cytoskeleton remodelling, invadopodia formation, and metastasis

ETs activate two receptor subtypes that belong to the G protein-coupled receptors (GPCR), known as ET_A_ receptor (ET_A_R) and ET_B_ receptor (ET_B_R), displaying 63 % similarity [1, 2]. The two receptors can be differentiated by agonists and antagonists binding affinity and in their cellular distributions. The ET_A_R binds ET-1 with the greatest affinity, whereas ET-1, ET-2, and ET-3 all have equal affinity for the ET_B_R. In human tissues alternatively spliced ET_A_R and ET_B_R transcripts have been reported, although the biological significance is still unclear. Epigenetic regulation is important also in the regulation of ET-1R in cancer. Silencing of the ET_B_R gene by DNA methylation was observed in different tumors, allowing to the downregulation of the receptor expression [6]. Recently, it has been shown that miR-30a functionally binds the ET_A_R 3’UTR, thereby inhibiting ET_A_R expression in ovarian cancer [7]. Moreover, posttranslational modifications, such phosphorylation, palmitoylation, and glycosylation, seem to be essential for the functional activity of both receptors [1, 2]. Based on the expression profiles of the ET-1R, there are cancers that predominantly express ET_A_R, such as ovarian, breast, colon, renal, gastric, pancreatic thyroid, and nasopharyngeal cancers; cancers expressing ET_B_R, such as melanoma, glioblastoma and astrocytoma, and those cancers expressing both ET_A_R and ET_B_R, such as bladder, lung, vulvar cancers and Kaposi’s sarcoma [2, 6]. ET-1R has been known for many years to regulate rapid signaling pathways through the involvement of G proteins, according with its nature of GPCR. In this canonical intracellular signaling, after agonist binding, ET_A_R couples to pertussin toxin-insensitive Gq/11 and Gq12/13 proteins, whereas ET_B_R couples to Gi/q and Gq/11 proteins [1, 2, 6]. Besides G-proteins, GPCR also activates a parallel set of intracellular pathways engaging with β-arrestin (β-arr) -1 or -2, scaffolding proteins that directly bind GPCRs recognizing the agonist-occupied receptor conformation [8, 9] (Fig. 1).

This review is divided into four parts. We first outline how ET-1/ET-1R axis actually underlies many features of cancers; in the second part, we review a large body of works on the specific role of β-arrs in different tumors; in the third part, we detailed the various β-arr-dependent signalings upon ET-1R activation in cancer. Finally, in the fourth part, we delineate the ET-1R cancer therapeutics capable to hamper β-arr-mediated signaling.

### Role of endothelin-1 signaling in cancer

Accumulating evidences demonstrated that activation of autocrine or paracrine signalings by ET-1R in cancer are complex regulatory mechanisms, and dysregulation of the ET-1R signaling is crucial for the development and progression of several human cancers [2, 10]. Indeed, ET-1R activation, through a G-protein dependent or β-arr-dependent mechanism, modulates different cancer-associated signaling pathways, including MAPK, NF-kB, β-catenin, PI3K/Akt and Rho GTPase, representing one of the major mediators of cell survival and proliferation, drug resistance, angiogenesis, osteogenesis, immune modulation, invasion and metastasis. Moreover, the complexity of ET-1R-mediated signaling is illustrated by its integrative crosstalk with non-GPCR driven signaling cascades, such as the epidermal growth factor receptor (EGFR), vascular endothelial growth factor receptor (VEGFR-2) or c-Src, creating further opportunities for therapeutic intervention [2, 10] (Fig. 1).

ET-1 is now considered responsible for the proliferation activity of different human cancer cells, acting alone or in cooperation with other tumor-related growth factors, favoring the activation of survival pathways [2, 6, 10]. ET-1-induced tumor promoting activities is also devoted to control migration and invasion, as well as epithelial-to-mesenchymal transition (EMT) and metastatic growth, most likely as consequence of a precise control over multiple signals, ranging from β-catenin signaling, EMT-related factors, integrin signaling as well as gap junction intercellular communication disruption [2, 10]. To invade and colonize targeted tissues, an ET-1R-driven effect involves also upregulation of expression, secretion and activation of several matrix metalloproteinases (MMPs), leading to enhanced pericellular protease activity [2, 10]. More recently, new studies on the interactions of ET-1R-driven pro-invasive pathways have highlighted that the mechanism by which cancer cells migrate through the extracellular matrix (ECM) is related to formation of invadopodia, specialized protrusions of the cell membrane of invasive tumour cells, rich in MMPs, facilitating their movement through the basement membrane and hence promoting tumour intravasation and metastasis [11, 12].

Strictly related to cancer progression and challenge in therapeutics, ET-1R is now recognized critically involved also in drug resistance and in the adaptive changes, like cancer stem cell (CSC) traits that, together with EMT, exacerbate metastatic spread and drug resistance [10]. In this context, in ovarian cancer ET_A_R overexpression correlates with platinum resistance, EMT marker expression and phenotypic changes consistent with stemness [13–16]. Similarly, high levels of ET-1 are expressed in CSC of colon cancer, thus underling the complex action exerted by ET-1R signaling in affording tumor cells invasive and metastatic properties, EMT, CSC-like phenotype, and drug resistance [2, 10].

Emerging data indicate also that ET-1R is capable of re-educating tumor-host environment, composed of immune cells, fibroblasts, and endothelial cells (EC), as well as growth factors, proangiogenic mediators, cytokines, chemokines, and other components of ECM, to form one that is “permissive”, in which multiple interactions between cancer cells and stroma determine not only tumor growth and metastasis but may also develop protective effects [2, 10]. The most studied and appreciated mechanism of ET-1 signaling in tumor microenvironment regards its involvement to direct angiogenesis and lymphangiogenesis through ET_B_R expressed on blood and lymphatic EC, and the critical interplay with hypoxia, maintaining constant angiogenic and pro-tumoral responses [2, 17–19]. Other studies demonstrated also integration between ET-1 axis signaling and cancer-associated fibroblasts or mesenchymal stromal cells in the formation of a supportive tumour stroma [2, 17, 18, 20]. An increased understanding of the mechanisms by which cancer cells escape the immune system recognizes now ET-1 axis as capable to mediate complex interactions between immune cells and malignant cells, acting on different subsets of immune effectors and regulatory cells, such as dendritic cells and tumor-associated macrophages [2, 10]. A critical interplay between ET-1 axis and T cell–infiltrated phenotype that might prevent T cell homing to tumor endothelium and hinders tumor immunotherapy has been shown in ovarian cancer and in gliomas [13, 21, 22], indicating that ET-1R has the potential to significantly change the ongoing immune response in the tumour microenvironment. In the bidirectional cross-talk between cancer cells and stromal cells, ET-1 signaling is implicated in a series of sequential and interrelated steps, also involved in chemoprotective signals [2, 23–25]. These findings extent previous findings regarding the role of ET-1 signaling in promoting metastasis [26–29], as recently reviewed [2, 10].

The role of ET-1R in the complexity of the tumor microenvironment interactions, and the importance of cell-cell interactions, led to evaluate its expression as potential biomarker in cancer diagnosis and treatment. Levels of ET-1 are higher in patients with bladder cancers, which are prone to metastasize, correlating with reduced patient survival, thus indicating ET-1 as a biomarker for lung metastasis [30]. Analysis of human ovarian cancer tissues demonstrated that ET_A_R overexpression is associated with worse survival, in particular in advanced stages and in the subset of platinum resistant cases [15]. However, many other studies need for optimizing the use of ET-1R to identify patients who are likely to respond to a given therapy, as well as for measuring patient response to therapy.

### Role of β-arrestin in cancer

Emerging data have revealed that β-arr recruitment represents a major non-G protein-dependent signaling pathway of GPCR, like ET-1R, in cancer. This function is largely related to the ability of β-arr to act as multi-functional adaptor capable to finely modulate multiple signaling pathways involved in tumor growth and metastasis, driven mainly by GPCRs (Table 1), but also by other non-GPCR pathways, such as transforming growth factor (TGF)-β, Hedgehog, Wingless, and Notch. Indeed, β-arr may directly interact with a plethora of proteins with various functions and localization, including nuclear proteins and cytoskeletal components [8]. One the best characterized signaling pathways mediated by β-arr is linked to non-receptor tyrosine kinase c-Src. Indeed, in different human cancer, such as prostate, colorectal, breast or gastric cancer, a complex and functional interaction between β-arr and c-Src driven by activation of GPCR, like prostanoid receptor 4, chemokine receptor-7 or GPR39, mediates the cross-talk with other several signaling, such as EGFR or Wnt signaling, representing additional mechanisms by which GPCR can sustain long-term biological responses [31–34]. The knowledge of the mechanisms activated by β-arr increased our understanding of its role to form and maintain signals, as an oncogenic hub, to direct cytoskeleton reorganization and to facilitate cancer cell migration induced by GPCR. β-arr function as signaling scaffolds to sequester regulators of actin assembly at the leading edge and to integrate multiple signaling molecules, fine tuning their signaling capacity in cell migration [35]. This regulation can occur either by scaffolding signaling proteins, such as Arp2/3 complex and WASP family of proteins or Rap1, RhoA and Ral GTPases, or by directly binding cytoskeleton proteins, such as proteins of cytoskeletal filaments, cytoskeletal accessory proteins, and motor proteins [35]. In agreement with these data, a global phosphorylation analysis of β-arr-mediated signaling identified a cytoskeleton reorganization network whereby β-arrs regulate phosphorylation of several key proteins [36, 37], during cancer cell migration and invasion.

**Table 1:** Roles of β-arrestins in human cancers

β-arr isoform	Tumor type	Role in cancer	References
β-arr1/β-arr2	Ovarian cancer	Chemoresistance, angiogenesis, migration, invasion, metastasis	[11, 15, 28, 29, 56, 58–61]
β-arr1	Lung cancer	Cell growth, migration, metastasis, stemness, angiogenesis Poor prognosis	[40–42, 54, 55, 62–66]
β-arr1/β-arr2	Prostate cancer	Cell viability and proliferation, chemoresistance, migration, metabolic alteration Regulator of androgen receptor expression	[32, 46, 48, 53, 66–68]
β-arr1	Acute lymphoblastic leukemia.	Cell growth, stemness Poor prognosis	[45, 69]
β-arr1/β-arr2	Chronic myeloid leukaemia	Cell growth, stemness Poor prognosis	[44, 70, 71]
β-arr1/β-arr2	Colorectal cancer	Cell growth, apoptosis, migration, invasion and metastasis	[33, 72–75]
β-arr1	Gastric cancer	Cell proliferation Poor prognosis	[34, 76]
β-arr1	Osteosarcoma	Proliferation and invasion	[77]
β-arr1/β-arr2	Breast cancer	Cell proliferation, migration and invasion and metastsis Multidrug resistance	[31, 36, 47, 50, 57, 78–86]
β-arr1	Neuroblastoma	Metastasis	[87]
β-arr1	Ewing's sarcoma	Drug resistance	[88]
β-arr1	Melanoma	Angiogenesis	[89]
β-arr2	Pancreatic cancer	Cell proliferation	[90]

Besides cytoplasmic functions, so far unexpected nuclear activities of β-arr are recently reported [38]. Thus, β-arr1 appears to regulate directly transcription causing nuclear interaction of β-arr1 with selected gene promoters and the recruitment of the histone acetyltransferase p300, thus enhancing local histone acetylation and gene transcription [39]. Following studies identified nuclear β-arr1 as a critical mediator of nicotine-induced human non-small cell lung cancer progression (NSCLC). Indeed, nuclear β-arr1/E2F1 complex on promoters of specific target genes drives proliferation, survival and EMT, as well as stemness [40–42]. Recently, it has been shown that β-arr1 contributes also to endothelial dysfunction promoted by nicotine, by promoting its recruitment to the E-selectin promoter as well as E-selectin expression, thus facilitating adhesion of monocytes to EC and endothelial damage, revealing a novel role for nuclear β-arr1 in the growth and metastasis of NSCLC [43]. Other studies depicted nuclear roles of β-arr1 in regulating the dynamic of gene expression in cancer cells by controlling the recruitment of epigenetic regulators, acting in histone modifications or DNA methylation [44–46]. More recently, it has been shown that nuclear β-arr1 play a critical role in facilitating adaptation of cancer cells to growth in conditions of metabolic alterations, called “pseudohypoxia”. Although it was already demonstrated that nuclear β-arr1 interacts with the primary factor mediating the response to low oxygen tension HIF-1α in breast cancer cells, a first genome wide-map of β-arr1 transcriptome in prostate cancer cells reported binding sites for β-arr1, p300 and HIF-1α on the regulatory regions of HIF-1α target gene promoter under hypoxic or pseudohypoxic conditions [47, 48]. This genomic landscape of β-arr1, revealing a partial overlap between β-arr1 and p300 binding sites, and the presence of non-overlapping sites, indicates that β-arr1 may also modulate transcription independently of p300, and also that exploring more in depth the nuclear function of β-arr1 might add one more layer of specialization of β-arr1 activities. The list of β-arr nuclear functions is growing very fast, and includes also new specific activities [49, 50]. Some of these specializations may also include an indirect way to control transcription, as demonstrated for a direct anti-apoptotic action of β-arr1 activated via β(2)-adrenoreceptors at the core of cell death machinery, in which nuclear β-arr1 facilitating activation of MDM2 and degradation of p53 promotes DNA damage accumulation [51]. On the other hand, the localization of several β-arr binding partners, such as JNK3, NF-kB and MDM2, are also regulated by ability of β-arr2 to retain them in the cytosol, and to change interactions with ubiquitinating and deubiquitinating enzymes, thus controlling crucial factors, such as p53, Wnt/β-catenin and FOXO1 [52, 53].

Using data from the Cancer Genome Atlas (TCGA) portal for β-arr1 expression across various tumor datasets, it has been shown that β-arr1 is amplified in various tumors, especially in high-grade ovarian cancer [10]. Interestingly, in patients with lung adenocarcinoma, the β-arr1 overexpression is an independent prognostic factor for tumor progression and unfavorable overall survival, suggesting a role of β-arr1 as promising biomarker to identify patients with poor prognosis [54]. In agreement with these findings, it has been demonstrated that in lung adenocarcinoma patients, co-expression of nuclear β-arr1 and p65 represents a novel predictor for worse prognosis [55]. Also, the higher expression of β-arr1 is positively correlated with clinical phases of chronic myeloid leukemia patients [44]. Given that the multidimensionality of β-arr confer highly advantageous signals to cancer cells, investigating whether β-arr can represent a prognostic factor will certainly emerge as the next challenge in the future cancer research studies.

### Multifunctional role of β-arrestin in ET-1 signaling in cancer

It has been shown that different GPCR signal through parallel pathways or support ligand bias to stimulate or promote different cellular responses, based on the ability of ligands to selectively activate G-protein- or β-arr-dependent signaling pathways. Considerable amount of data have been accumulated showing that β-arr1 plays a major role in tumor-promoting effects of ET-1R signaling in cancer [2, 10]. These studies highlighted the formation of new ET-1R-driven β-arr-mediated multiprotein signaling complexes, known as a “signalosomes,” in which β-arr acts as cytosolic or nuclear scaffold, adaptor, and signal transducer, orchestrating a variety of fine-tuned intracellular signaling pathways than culminate in sequential steps of tumor progression. Recently, new roles for β-arr1 have emerged in ET-1 signaling, sequestering and bringing the right players for actin assembly activities and upstream regulators at the leading edge to drive local invasion and metastasis [11, 56]. Thus, ET_A_R/β-arr1 signaling represents a novel driver of cytoskeletal organization and Rho GTPase activity supporting cell motility. In ovarian cancer cells, ET-1 through β-arr1 activates RhoA GTPase and, downstream, Rho-associated coiled coil-forming kinase 1 (ROCK1) kinase activity, leading to enhanced cell migration and protrusion formation. Most importantly, it has been revealed that β-arr1 acts as signal-integrating module of Rho GTPase activity for specific formation and maturation of invadopodia, small invasive protrusions of localized protease activity, which are formed in response to signaling events leading to dynamic branched actin assembly at membrane sites and are cellular hotspots for secretion of proteinases degrading ECM. This is accomplished by the direct interaction of β-arr1 with a Rho guanine nucleotide exchange factor (GEF), PDZ-RhoGEF, which activates Rho GTPase proteins by catalyzing the exchange of GDP for GTP. Interestingly, β-arr1/PDZ-RhoGEF interaction mediates ET_A_R-driven ROCK-cofilin pathway through the control of RhoC activity. This novel interaction is functional to invadopodia formation and activation of key invadopodia proteins, cortactin, cofilin and membrane type I (MT1)-MMP. Collectively, these data unveiled a noncanonical activation of the RhoC/ROCK pathway through the β-arr1/PDZ-RhoGEF complex, establishing ET-1R/β-arr1 axis as a novel regulator of invadopodia protrusions during tumor invasion [11]. These results are in line with previous findings demonstrating the role of β-arr in controlling the cofilin pathway localizing actin to the leading edge of cells to promote invasive protrusions during tumor cell migration [12, 35, 57].

**Fig. 2:**
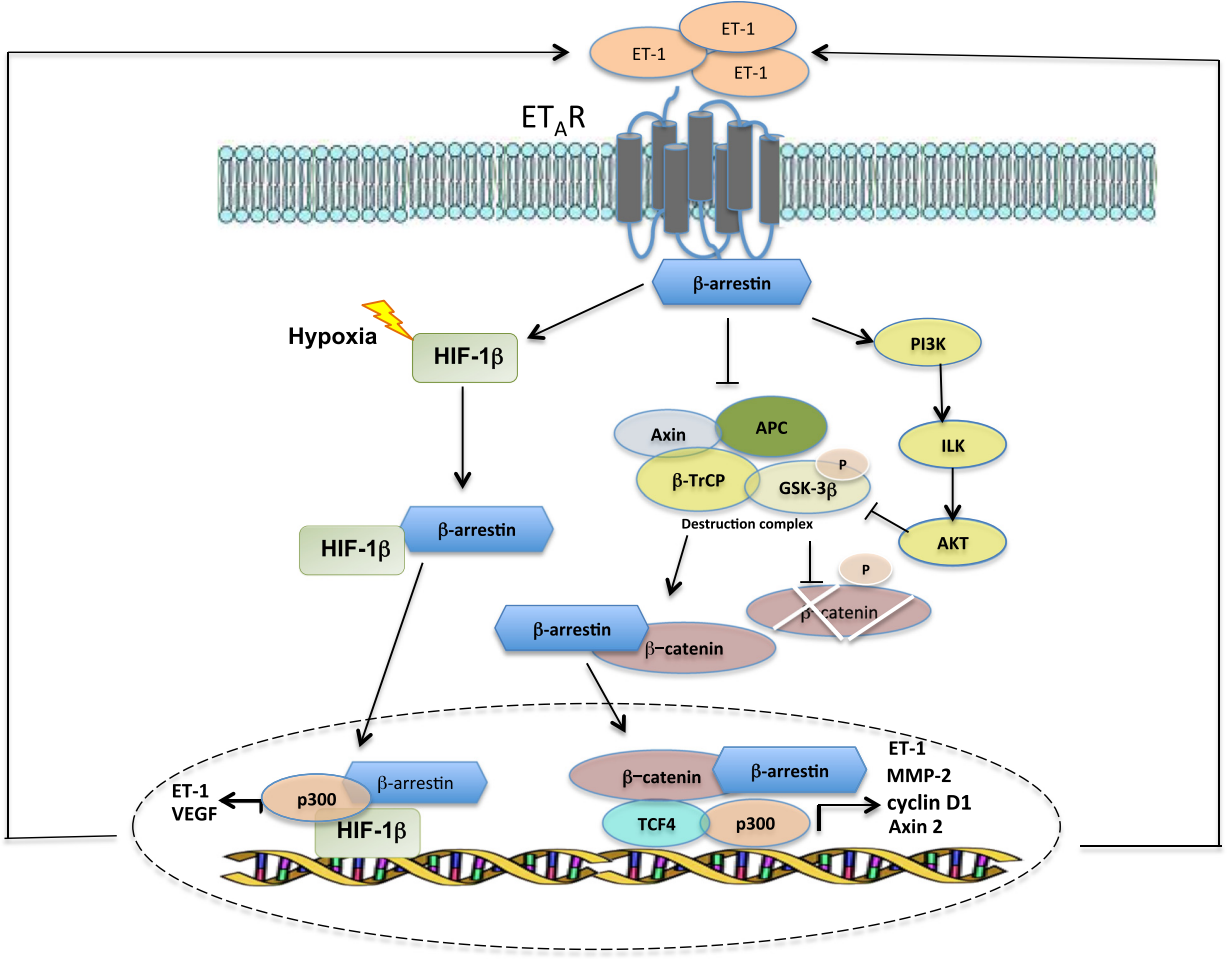
Nuclear function of β-arrestin1 in ET-1 signaling. Binding of ET-1 to ET_A_R leads to the recruitment of β-arr1 to the activated receptor, which mediates the phosphatidylinositol 3-kinase (PI3-K)/integrin linked kinase (ILK)/Akt signaling route, which causes the phosphorylation and inactivation of glycogen synthetase kinase 3β (GSK3-β) and accumulation of a non-Ser/Thr phosphorylated, active β-catenin. In parallel, ET_A_R/β-arr1 complex binds axin, thereby promoting the release of GSK-3β from the β-transducin repeat containing protein (β-TrCP) and adenomatous polyposis coli (APC)-mediated degradation machinery, and its inactivation. In turn, β-arr1 shuttles with β-catenin to the nucleus and by interacting with p300 histone acetyltransferase enhances β-catenin/T-cell-specific transcription factor-4 (TCF4) activation, thus promoting the transcription of genes, such as ET-1, matrix metalloproteinase 2 (MMP-2), cyclin D1 and Axin 2, leading to enhanced cell motility and aggressiveness. Moreover, in ET-1-dependent manner, β-arr1 shuttles into the nucleus, where it interacts with HIF-1α to form a transcriptional complex with p300 required for histone acetylation and for the transcription of HIF-1α target genes, such as ET-1 and VEGF, a mechanism that can be amplified by hypoxia. Both these signals lead to the amplification of the ET-1 autocrine loop

Different studies demonstrated that the recruitment of β-arr to ET_A_R represents a checkpoint controlling pathways converging on β-catenin signaling to promote ovarian cancer invasion and metastasis. Indeed, after binding of ET-1 to ET_A_R, β-arr1 and β -arr2 were recruited to the receptor, to form at least two trimeric signaling complexes or “signalplexes”: one through the interaction with c-Src leading to EGFR transactivation and, downstream, β-catenin tyrosine phosphorylation, and the another through the physical association with axin, contributing to release and inactivation of glycogen synthase kinase-3β and β-catenin stabilization, both concurring to activate β-catenin signaling pathways, and to activate transcription of genes involved in EMT, invasion and metastasis [28]. Most important, in xenograft models of intraperitoneal ovarian cancer metastasis, cells expressing a mutant β-arr ineffective to bind c-Src show reduced metastatic ability, and reduced expression of active β-catenin is observed in the metastatic nodules [28]. Of clinical relevance, ET_A_R is expressed in 85 % of ovarian cancer and is preferentially co-expression with β-arr in the advanced tumors, further revealing the pathophysiological role of β-arr1 and ET-1R in tumor progression [28]. To underscore the complexity of the mechanisms available to ET_A_R to interlink β-catenin pathways, it has been explored whether β-arr1 may also provide a link between ET_A_R and β-catenin transcription as nuclear scaffolding mediating epigenetic modification [29]. The mechanism underlying ET-1-driven final transcriptional output of β-catenin includes ET_A_R-dependent β-arr1 nuclear translocation, direct binding to β-catenin and their nuclear accumulation (Fig. 2). β-arr1/β-catenin interactions, by favouring the dissociation of histone deacetylase 1 and the recruitment of p300 on β-catenin target gene promoters, such as ET-1, AXIN2, MMP2 and cyclin D1, result in enhanced gene transcription, required for cell migration, invasion and metastasis. Thus, silencing of β-arr1 or nuclear mutant β-arr1 expression inhibits ovarian cancer metastasis [29]. The findings of in vivo direct β-arr1/β-catenin association at specific β-catenin target gene promoters derived from analysis of human ovarian cancer tissues, correlating with tumor grade, unravel new components in the role of β-arr1 as critical nuclear mediator in cancer [29]. Additional studies identified β-arr1 as a co-pilot of ET_A_R to organize complex signaling networks also controlling EMT phenotype, stemness features and drug resistance, expanding previously knowledge about the chemoresistance-associated functions of ET_A_R. Indeed, ET_A_R/β-arr1-driven epigenetic modifications and β-catenin transcription sustain chemoresistance onset by enhancing transcription of genes, such as ET-1, linked to chemoresistance [15]. The general idea is that the spectrum of pathways and interactions through which β-arr1 might regulate cancer invasion, metastasis, and chemoresistance, is more then until now demonstrated. Indeed, in melanoma cells, the cytosolic scaffold function of β-arr1 is also necessary to activate crosstalk with VEGFR-2, promoting melanoma cell plasticity, motility, and neovascularization [10].

More recently, a novel mechanism of β-arr1 nuclear function in ET-1R signaling involving HIF-1α activity has been described (Fig. 2). In ovarian cancer cells, activation of ET_A_R, by mimicking hypoxia, induces the nuclear β-arr1/HIF-1α interaction and the recruitment of p300 to hypoxia response elements of gene targets, resulting in enhanced histone acetylation, transcription and thus release of HIF-1α targets, such as ET-1 and VEGF, required for tumor cell invasion and pro-angiogenic effects in ECs [58]. These results highlight the presence of a self-amplifying HIF-1α-mediated transcription of ET-1 sustaining a regulatory circuit involved in invasive and angiogenic behaviours. These findings broaden previous data in prostate and breast cancer [47, 48]. The intriguing role of nuclear β-arr1 was further amplified by other findings revealing a previously unrecognized pathway that depends on β-arr1 to sustain NF-kB signaling in response to ET_A_R activation [59]. Collectively, these multiple lines of evidence underscore more appealing aspects of β-arr1 as transducer of ET-1 signaling during cancer progression, including cytoskeletal organization.

All these findings suggest that further investigations are required to elucidate the role and regulation of these metastatic promoting functions driven ET-1R signaling, to determine the degree of which is dictated by the G-protein-dependent and β-arr dependent signaling as well as the involvement of β-arr isoforms, that will aid in the identification of new targets for the design of novel therapeutics against cancer. In this regard, in ovarian cancer model it has been clearly depicted the functional selectively of β-arr-dependent signaling driven by ET-1R [10, 60], that promotes biological responses differing quantitatively and qualitatively in promoting tumor progression. Thus, β-arr1 responds to ET-1R activation reflecting changes in the dynamic of actin cytoskeleton, which appears to be crucial to integrate multiple signals, setting β-arr functions as an absolute requirement for cancer signaling.

### Clinical potential of ET-1R therapeutics controlling β-arrestin signaling

ET-1R is now considered one of the most attractive therapeutic targets in cancer, representing one of the best-characterized systems with respect to functional consequences of GPCR–β-arr interactions. Different findings reveal that ET_A_R/β-arr interaction is fully engaged in transmitting signals from the membrane to nucleus, promoting early and late events leading to tumor progression. These data illustrate also the utility in the translation of the concept of seeking antagonists with functional selectivity respect to G-protein-independent and β-arr-dependent ET-1 signaling to block cellular responses associated with tumor progression [10]. Considering the effects of β-arr as both cytoplasmic and nuclear messengers in tumor invasion and metastasis as well as in chemoresistance, the translation from basic biology to clinical drug development might consider that disruption of the ET_A_R/β-arr interaction can impair EMT, invasion, metastasis and chemoresistance, suggesting a possible avenue for therapeutic intervention. A plethora of studies in different human cancer highlighted the efficacy of targeting ET_A_R in vitro and in preclinical models, in which ET_A_R blockade with different ET_A_R-selective antagonists (atrasentan and zibotentan) results in antitumour activity, by concomitant growth inhibition and apoptosis induction, an effect that can be potentiated from combined treatment of chemotherapeutics or targeted therapy [2, 10]. The efficacy of this target therapy is extended to control also drug-resistance and metastasis, as shown in different tumor models [2, 10]. However, the failure of clinical trials using selective antagonists and the screening of antagonists tested for their ability to alter ET-1-driven cancer cell growth and progression, uncovered the dual ET_A_R/ET_B_R antagonist as a promising drug for treatment of ET-1R-driven tumors. Macitentan is a non-selective and dual ET_A_R/ET_B_R antagonist, approved for the treatment of pulmonary hypertension [10]. Preclinical data in different tumor models showed drug efficacy in controlling tumor growth and metastasis, in monotherapy or in combination with others drugs, such as chemotherapeutic drugs, predicting clinical success [2, 10]. According with a recurring theme in the utilization of molecularly targeting therapeutics that should target both tumor cells and microenvironment where tumor cells thrive, macitentan simultaneously targets cancer cells, which typically express ET_A_R, and stromal elements through ET_B_R, enhancing antitumour immune and anti-angiogenic mechanisms. Moreover, considering the decisive role of ET-1 signaling in the physical interactions of cancer cells and tumor-associated stromal cells, to sustain cancer metastasis as well as to act as chemoprotective mediator, this kind of drug represent a promising therapeutic opportunity [15, 24–27]. Mounting evidence suggests that drugs, like macitentan, serve as effective tools for cancer therapy also for the ability to selectively targeting specific β-arr-dependent downstream signaling [10]. In ovarian cancer, where it has been dissected the role of β-arr in ET-1 signaling, in vivo, silencing of β-arr1 or macitentan treatment, impairing the signaling pathways involved in cell survival, EMT, and invasion, reduces tumor growth, vascularization, intravasation, and metastatic progression, and potentiates the cytotoxic effects of cisplatinum [15, 16]. Continuing efforts to fully understand the biological functions and associated mechanisms of ET-1R will likely facilitate the development of new targets and innovative pharmacological strategies for treating cancer patients. Further investigations will hopefully drive preclinical validated antagonists into clinical studies where their true therapeutic efficacies against cancer vulnerability can be evaluated.

## Conclusions

β-arr is a well-known primary effector of GPCR pathway, crucial in tumor growth and progression. Increasing evidences have provided insights into the regulation of this protein and its mechanism of action. The findings highlighted in this review offer a broad overview of the biological activity elicited by ET-1R/β-arr in tumorigenesis and metastasis progression. The above information indicates that ET-1/ET-1R axis activates through β-arr1 many signals on epigenetic inputs that sustain ET-1R activities in cancer and tumor microenvironment. Cancer cells may use different cytoplasmic and nuclear roles of β-arr1 in a manner that may change between different tumors and even into tumoral lesions. Thus an important aspect that deserves attention is the mechanism by which nuclear β-arr1 selects transcriptional partners, revealing new players by which ET-1R/β-arr orchestrate cancer cell behaviour and outcomes. A better understanding of these events may suggest therapeutic antitumoral strategies, for example focusing on the ET-1R antagonist currently used in clinical practice, allowing precise control over signaling pathways. Clearly, being able to block ET-1R and turn-out the β-arr functions would represent a new era for ET-1-therapeutics to be exploited for cancer treatment.

## Abbreviations

CLL, chronic lymphocytic leukemia; CSC, cancer stem cell; EC, endothelial cells; ECM, extracellular matrix; EGFR, epidermal growth factor receptor; EMT, epithelial-to-mesenchymal transition; ET, Endothelin; ET-1R, endothelin-1 receptor; ET_A_R, ET_A_ receptor; ET_B_R, ET_B_ receptor; FOXO1, forkhead box protein O1; GEF, Rho guanine nucleotide exchange factor; GPCR, G protein-coupled receptors; HIF-1, hypoxia-inducible factor; miRNA, microRNAs; MMPs, matrix metalloproteinases; MT1-MMP, membrane type I; NFkB, nuclear factor kappa B; NSCLC, non-small cell lung cancer progression; ROCK1, Rho-associated coiled coil-forming kinase 1; TCGA, The Cancer Genome Atlas; VEGFR-2, vascular endothelial growth factor receptor-2; β-arr1, β-arrestin1
